# Using Cerebral Metabolites to Guide Precision Medicine for Subarachnoid Hemorrhage: Lactate and Pyruvate

**DOI:** 10.3390/metabo9110245

**Published:** 2019-10-23

**Authors:** Kaneez Zahra, Neethu Gopal, William D. Freeman, Marion T. Turnbull

**Affiliations:** 1 Department of Neurology, Mayo Clinic, 4500 San Pablo Rd, Jacksonville, FL 32224, USA; 2 Department of Neurologic Surgery, Mayo Clinic, 4500 San Pablo Rd, Jacksonville, FL 32224, USA; 3 Department of Critical Care Medicine, Mayo Clinic, 4500 San Pablo Rd, Jacksonville, FL 32224, USA

**Keywords:** subarachnoid hemorrhage, cerebral metabolism, lactate, pyruvate, lactate-pyruvate ratio, delayed cerebral ischemia, vasospasm, precision medicine

## Abstract

Subarachnoid hemorrhage (SAH) is one of the deadliest types of strokes with high rates of morbidity and permanent injury. Fluctuations in the levels of cerebral metabolites following SAH can be indicators of brain injury severity. Specifically, the changes in the levels of key metabolites involved in cellular metabolism, lactate and pyruvate, can be used as a biomarker for patient prognosis and tailor treatment to an individual’s needs. Here, clinical research is reviewed on the usefulness of cerebral lactate and pyruvate measurements as a predictive tool for SAH outcomes and their potential to guide a precision medicine approach to treatment.

## 1. Introduction

Subarachnoid hemorrhage (SAH) is one of the deadliest types of stroke [1]. It comprises 2% to 5% of all strokes, amounting to an estimated 30,000–46,000 people per year in the US, with a mortality rate of nearly 40% within the first 30 days of hemorrhage [2]. Patients with SAH commonly present with a sudden onset of severe headache, colloquially described by the patient as the “worst headache of their life.” SAH is caused by bleeding into the subarachnoid space from a ruptured aneurysm (aSAH), arteriovenous malformation, or head injury [3].

Patients suffering from SAH face both immediate [4,5] and delayed risks of neurological injury [6,7]. Intracranial bleeding and blood breakdown products cause early brain injury by inducing widespread inflammation, ionic imbalance, oxidative stress and hypoxia within the first 72 h [6,8]. Over the following 21 days, SAH blood creates a delayed sterile inflammatory response that results in narrowing of the arterial vessel lumen and renders the vessel largely unreactive. This delayed effect to intracranial arteries occurring after SAH is called vasospasm which can be seen on a cerebral angiogram and can cause additional brain damage by restricting the flow of blood to downstream brain tissue [9,10]. Delayed cerebral ischemia (DCI) is another major contributor to poor patient prognosis after SAH and can result in severe neurological deficits (e.g., aphasia, hemiparesis) [11]. DCI can occur in conjunction with angiographic narrowing of vessels or may occur independently, [12] and can trigger further injury mechanisms, such as spreading depolarizations (SDs) [13]. SDs are slowly propagating waves of depolarization associated with near-complete failure of brain ion hemostasis, which results in cellular injury, altered energy metabolism and changes in cerebral blood flow [13].

Predicting if, or when, a patient with SAH will develop vasospasm or DCI remains problematic since the symptoms may occur suddenly, without warning and leave little time for therapeutic intervention. Furthermore, despite continuous therapy with nimodipine, patients are still at risk for vasospasm, DCI, and SDs, thus an individualized medicine approach based on the molecular profiles of brain injury could guide therapeutic intervention and lead to better patient outcomes. An analysis of different metabolites produced by the brain in response to acute injury has been suggested to gauge disease severity and predict patient outcomes after SAH. More recently, the development of omics technology which can simultaneously measure thousands of molecules (e.g., metabolomics for analysis of metabolites) has been used to search for new targets in many disease states. However, the field of SAH research is still only beginning to explore the potential of broad metabolomic profiling of patients [14,15,16]. Instead, more targeted, hypothesis-driven research investigating changes in metabolites after SAH has been conducted.

Lactate and pyruvate are biochemical intermediates of cellular energy production which have been widely investigated as potential injury biomarkers for SAH. The changes in lactate and pyruvate levels have also been described in a range of neurological injury states such as traumatic brain injury [17,18], ischemic stroke [19], and neurodegenerative disease [20]. Under normal conditions, lactate is produced from two major sources: astrocytes and neurons. Lactate is used as fuel to drive the production of adenosine triphosphate (ATP). In astrocytes, glucose from blood or stored glycogen is mobilized to generate lactate when neuronal energy demands increase [21,22]. The astrocyte produced lactate is then transported to neurons via the astrocyte-neuron lactate shuttle, where it is converted into pyruvate by neurons to produce ATP via oxidative phosphorylation [23]. This feeding of lactate from astrocytes to neurons can help balance the metabolism of glucose in the brain [24].

**Figure 1:**
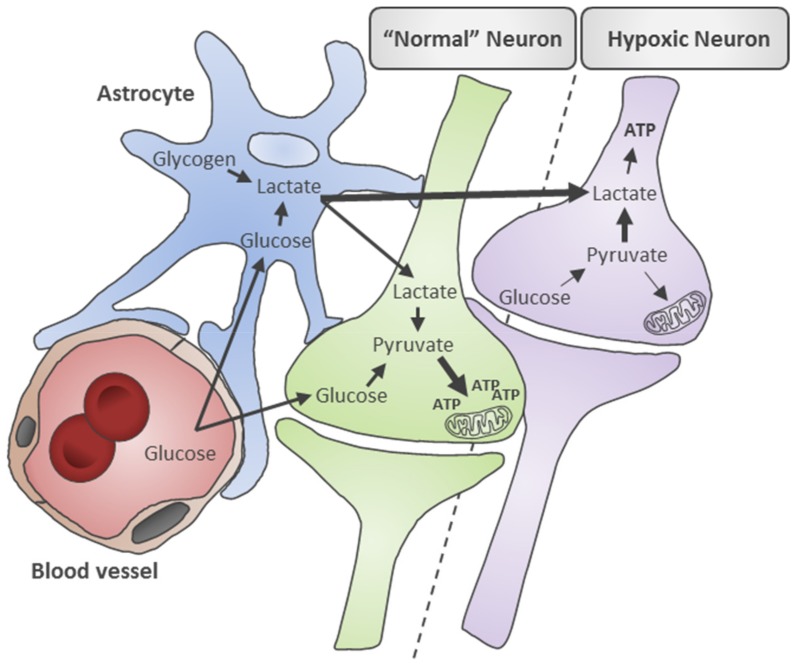
Schematic of lactate and pyruvate metabolism in neurons and astrocytes under normal and hypoxic conditions. Under physiologically normal conditions, glucose is transported from blood vessels across the blood-brain barrier to neurons and astrocytes. In neurons (green cell), glucose is converted into pyruvate by aerobic glycolysis which ultimately generates adenosine triphosphate (ATP) in mitochondrial. In astrocytes (blue cell), glucose and stored glycogen make lactate which is then shuttled to neurons via the astrocyte-neuron lactate shuttle where it is used as fuel to make ATP. Under hypoxic conditions, glucose supply to neurons (purple cell) is decreased and the absence of oxygen causes pyruvate metabolism to shift towards lactate production and less efficient ATP production to meet cellular energy demands. Lactate production and supply by astrocytes is also increased.

Alternatively in neurons, glucose that enters the cell is converted into pyruvate and then converted into either acetyl coenzyme A (acetyl-CoA) or into lactate, depending on the presence or absence of oxygen [25]. Under aerobic conditions, pyruvate produced through cytosolic glycolysis is converted into acetyl-CoA by the enzyme complex pyruvate dehydrogenase (PDH) then enters the mitochondrial citric acid cycle to produce ATP. In anaerobic conditions such as hypoxia or ischemia, oxidation cannot occur and the accumulation of cofactors results in the inhibition of PDH activity. The conversion of pyruvate into acetyl-CoA halts and is instead shifted towards conversion into lactate which allows for continued anaerobic ATP synthesis however at a fraction of aerobic ATP production (Figure 1) [25]. Consequently, cerebral lactate and pyruvate levels provide important readouts of the relative contributions of aerobic and anaerobic metabolism to brain energy production [26]. High cerebral lactate levels may also reflect cerebral hyperglycolysis (increased glucose metabolism) initiated as part of a stress response when substrate delivery does not keep up with extreme energy demands, such as after SAH. Hyperglycolysis is reflective of an attempt to restore energy supply/demand by utilizing more glucose, which leads to elevated lactate and pyruvate levels [27].

Following a cerebral hemorrhage, the reduced blood flow and subsequent ischemia or hypoxia causes perturbations in brain energy production. This review aims to summarize how changes in brain energy production, represented as the changes in the levels of lactate, pyruvate, and the lactate-pyruvate ratio (LPR), have been shown to reflect the extent of injury after SAH, and can correlate with clinical events and patient outcomes. Lactate and pyruvate concentrations can be measured from cerebral fluids, such as cerebrospinal fluid (CSF) and interstitial fluid (ISF), or from patient blood. CSF is often sampled by lumbar puncture or external ventricular drain (EVD) which is placed in patients to alleviate intracranial pressure (ICP) by diverting CSF flow. ISF is sampled using cerebral microdialysis (CMD) and can be more reflective of local changes after injury as it can be sampled from areas adjacent to, or at, the lesion site. The measurement of lactate and pyruvate levels can potentially predict the occurrence of secondary events, such as vasospasm and DCI, and ultimately may guide targeted therapeutic management of patients with SAH in an effort to reduce mortality and morbidity.

## 2. Metabolic Mechanisms Underlying Disturbed Energy Metabolism in SAH

To characterize brain lactate metabolism in the context of SAH, Oddo and colleagues set out to determine if the elevations in cerebral lactate occur as a consequence of hyperglycolysis or hypoxia [28] (summarized in Table 1). To study this, they measured interstitial lactate and pyruvate by CMD, in combination with brain oxygen (PbtO_2_) monitoring in patients with poor grade SAH. They categorized 31 patients with elevated lactate (>4 mmol/L) into three groups: hypoxic (PbtO_2_ < 20 mmHg); non-hypoxic and hyperglycolytic (pyruvate > 119 µmol/L); non-hyperglycolytic. They found that the elevations in interstitial lactate were more often attributable to cerebral hyperglycolysis (78% of patients) than brain hypoxia (11% of patients) and that cerebral hyperglycolytic lactate was a predictor of a good 6-month outcome (modified Rankin score, mRS of 0–3). Moreover, mortality was more associated with elevated lactate and brain hypoxia [28].

This was followed by a study exploring whether biochemical changes in SAH patients exhibit patterns relating to mitochondrial dysfunction or to cerebral ischemia after SAH [29]. Mitochondrial dysfunction was characterized by an increased LPR (>30) due to the increase in cerebral lactate with normal or elevated pyruvate levels (>70 µM), whereas cerebral ischemia was characterized as an increased LPR (>30 and >40) due to an increase in cerebral lactate but with a pronounced decrease in cerebral pyruvate (<70 µM). Interstitial lactate and pyruvate were collected by CMD in 55 patients with severe SAH (Fisher grade 3 or 4). They found biochemical patterns of mitochondrial dysfunction in 29 patients and cerebral ischemia in 10 patients, including 6 patients who also demonstrated periods of mitochondrial dysfunction. They concluded that mitochondrial dysfunction is a common cause of metabolic crises and disturbed energy metabolism in patients with SAH and not always associated with a reduction in cerebral blood flow or tissue oxygenation [29].

## 3. Lactate and Pyruvate after SAH: Predictors of Clinical Events and Outcomes?

To determine if initial serum lactate (measured during the first 12 h of admission) can be used as an objective predictor of mortality, a large retrospective review of 249 SAH patients was conducted. When controlling for the admission Hunt and Hess grades, Aisiku and colleagues found that elevated serum lactate levels (3.5 ± 2.5 mmol/L versus 2.2 ± 1.6 mmol/L) was predictive of patient mortality, with over half of the deaths occurring within 48 h [30]. This is in contrast to another study where SAH patients that showed that elevated serum lactate (defined as > 2.2 mmol/L based on laboratory normals) measured within 24 h of admission, was not independently predictive of patient mortality or discharge disposition [31]. Moreover, admission lactate was not associated with the development of vasospasm or DCI [31]. This disparity in the findings may be due to use of a different cut off value for elevated lactate (3.5 mmol/L versus 2.2 mmol/L).

The changes in metabolite levels have also been used to predict the outcome beyond the binary of mortality/survival. Cerebral metabolism in patients with low grade SAH (World Federation of Neurological Surgeons, WFNS score 1 to 3) was compared to patients with high grade SAH (WFNS score 4 or 5) by a microdialysis catheter inserted into the vascular territory of an aneurysm following clip placement. The lactate levels and LPR was significantly elevated in high grade SAH patients compared to low grade SAH patients, however, only LPR (mean LPR over first 3 days) was predictive of poor patient outcomes (severe disability, vegetative state, or death) at 12 months [32]. Similarly, in a study conducted in 10 aSAH patients, ISF lactate and other cerebral metabolites were analyzed for 4.6 ± 0.5 days (up to 9 days after ictus) to determine if they correlated with patient outcome. They observed that patients with unfavorable outcomes at three months (Glasgow outcome score, GOS, score of 1–3) primarily associated with the development of large infarctions and had a 10-fold increase in lactate levels compared to patients with favorable outcomes (GOS of 4 or 5) [33]. This data suggests that lactate is useful as a parameter for estimating the extent of cerebral injury in aSAH patients [33]. A recent untargeted metabolomic analysis of CSF in SAH patients also identified enhanced pyruvate metabolism in patients with a Hunt and Hess grade over III. Moreover, the elevated levels of pyruvate in the CSF were significantly associated with poor grade SAH (WFNS scale > III) [16]. Interestingly, Li and colleagues found no significant associations with lactate, and that different amounts and distribution of bleeding did not correlate with significant metabolome variation in patient CSF [16].

Similarly, changes in cerebral metabolism have been measured to determine if they can be predictive of detrimental secondary events. Cerebral microdialysis was performed at the patient bedside in one study to investigate if the changes in lactate and pyruvate levels could predict impending regional cerebral ischemia [34]. Patients with aSAH were classified according to clinical presentation and microdialysis samples were taken hourly. Patients with acute neurological deficits had a significantly higher LPR in the first week post SAH, compared to asymptomatic patients or patients with delayed neurological deficits. Correspondingly, patients with delayed neurological deficits had higher cerebral ISF lactate levels on days 1 to 8, along with a higher LPR on days 3 to 8 post-SAH, compared to asymptomatic patients. An increase in lactate was identified as the earliest marker of impeding ischemia and the development of cerebral infarction in patients with SAH, followed by a rise in LPR [34]. Likewise, patients with poor grade SAH (Glasgow Coma Score, GCS, ≤ 8) who had ISF sampled for up to 10 days after SAH, had a significantly higher LPR (51 ± 36) than those who did not develop DCI (31 ± 10) [35]. Similarly, a prospective study of 18 patients demonstrated that interstitial lactate at day 7 was higher in patients with delayed ischemic neurological deficits compared to those with no deficits [36].

The microtubule-associated protein tau has been studied as a surrogate marker for axonal injury in patients with SAH [37]. Studies have shown that that this biomarker has the potential to stratify the severity of SAH and potentially serve as an early warning sign for DCI [37,38]. To determine if cerebral tau levels correlated with the levels of lactate and pyruvate after SAH, serial CMD samples were collected from 22 aSAH patients and assessed for total tau protein, lactate, and pyruvate [39]. They found that tau protein positively correlated with lactate, pyruvate, and LPR, and that tau protein levels were elevated during episodes of hypoxic (PbtO_2_, < 20 mmHg) lactate elevation (> 4 mmol/L). Moreover, high tau levels were associated with poor functional outcomes (mRS ≥ 4) at 12 months after SAH with adjustment for disease severity and age. Similarly, the elevated levels of tau were indicative of impaired cognition, visual conceptualization and frontal executive functions at 1 year after SAH [39].

Lactate levels measured from serum have also been tested to see if they correlate with the patients who went on to develop DCI and poor outcomes [40]. Patients who developed DCI had significantly higher serum lactate levels during the first 24 h after admission than patients without DCI (2.1 mmol/L versus 1.5 mmol/L) and had poorer outcomes (mRS of 4–6 at 3 months). This data suggests that routinely available laboratory tests may help identify the patients at risk for impending complications and poor neurological outcomes [40]. This was further expanded by Okazaki and colleagues who performed serial blood lactate measurements every 6 h from SAH patients in the intensive care unit (ICU) and correlated them with neurological outcomes. They found that the blood lactate levels at 48 h of admission was the most accurate predictor of a poor outcome (mRS of 3–6), with a cut off value of 1.1 mmol/L of lactate (sensitivity, 40%; specificity, 92.1%) [41].

CSF levels of lactate have also been investigated to determine if they associate with age, treatment after SAH, clinical condition, and cerebral perfusion. CSF lactate levels were obtained from EVD catheters 0–240 h after SAH in 33 patients and elevated CSF lactate levels (defined as > 2.1 mmol/L) were associated with patients 61 years old or greater, and in patients treated with endovascular coiling but not surgical clipping. However, no association was observed between elevated CSF lactate levels and cerebral perfusion or neurological impairment [42]. The association between higher age and elevated CSF lactate has previously been reported in a normal population and is considered a biomarker of aging [43].

A study done by Mori and colleagues showed that daily monitoring of CSF lactate from an EVD for 12 days after SAH served as a biomarker to predict the occurrence of vasospasm. They demonstrated that elevated levels of CSF lactate, along with a high LPR on day 5 to 7, correlated with the onset of cerebral vasospasm [44]. This is in contrast to Renfrow and colleagues who investigated the utility of CSF lactate, measured from EVDs placed for hydrocephalus relief in aSAH patients, for predicting vasospasm and outcomes. Although they did not observe a correlation between CSF lactate levels and symptomatic vasospasm, they report that it correlated with intraventricular hemorrhage extension and unfavorable outcomes at discharge, defined as discharge to a nursing home, long-term acute care, hospice, or death before discharge [45]. CSF lactate also correlated with admission severity grades, such as Fisher’s grade and Hunt and Hess scale [45].

Similar to vasospasm and DCI, the occurrence of SDs after a stroke affect the levels of lactate and pyruvate in the brain. The restoration of ionic gradients after SDs is metabolically challenging for the brain, requiring the rapid consumption of glucose to correct the near complete loss of membrane potential [46,47]. Consequently, glucose utilization increases dramatically with a corresponding rise in lactate levels and this can be further exacerbated by subsequent, consecutive SDs which double and triple the utilization of glucose in an already injured brain [46,48,49].

## 4. Brain Hemodynamics and Lactate and Pyruvate

The local release of lactate has been reported to regulate vascular tone through multiple mechanisms [57,58,59,60]. To explore the relationship between intracranial hemodynamics and cerebral energy metabolism in the context of SAH, the levels of cerebral lactate and pyruvate have been assessed and correlated to the measures of cerebral pressure and blood flow in SAH patients. In one study, the interstitial levels of lactate and pyruvate were measured in 33 SAH patients in the ICU. They observed that pyruvate was positively correlated with cerebral perfusion pressure (*r* = 0.24) and mean arterial blood pressure (*r* = 0.23). ICP periods of ≤ 10 mmHg were also significantly associated with higher pyruvate levels (mean, 148 µM versus 143 µM), lower lactate levels (mean, 3.6 µM versus 4.1 µM) and lower LPR (mean, 25 versus 29) compared to periods of ICP over 10 mmHg. Manipulation of ICP, such as opening the EVD to reduce pressure in patients with high ICP, resulted in a surge of pyruvate [61]. Similarly in other studies, increased ICP (≥ 20 mmHg) on days 1 to 7 after SAH was positively correlated with an elevated ISF LPR (> 25) [62] and decreased cerebral perfusion [50]. The derangement of metabolites occurred prior to the increase in ICP and lowered cerebral perfusion, with over 80% of patients with intracranial hypertension presenting with deranged metabolism (an LPR of greater than 25) prior to the first rise in ICP. LPR had a significant influence on poor 12 month GOS, and high ICP (> 20 mmHg) and high LPR were strong predictors of death [51,62].

Rostami et al. studied cerebral blood flow (CBF) and interstitial metabolites including lactate in 30 patients with severe SAH (modified Fisher grades of 3 and 4) in an effort to predict which patients would go on to develop DCI. They monitored CBF and interstitial metabolites by microdialysis for the first 3 days after hemorrhage, and found that the patients who went on to develop DCI had lower global and regional CBF (global CBF, 23.7 ± 6.7 mL/100 g/min versus 37.5 ± 13.7 mL/100 g/min and regional CBF, 21.4 ± 5.3 mL/100 g/min versus 34.4 ± 12.8 mL/100 g/min) and elevated lactate levels compared those who did not (4.8 ± 2.2 mmol/L versus 3.4 ± 1.7 mmol/L). No other metabolite measured showed a significantly different change between the DCI and no-DCI groups, and they did not see a significant rise in LPR [52]. Another study done by Rostami and colleagues showed an equivalent correlation between low CBF and elevated interstitial lactate in the same vascular area of a patient’s brain during the acute phase (days 0–3) following SAH (*r* = 0.911) [53].

## 5. Association of Lactate and Pyruvate with Secondary Complications and Events

The association between the changes in lactate and pyruvate levels with secondary events and complications, such as consciousness, pneumonia, edema, and hydrocephalus, after SAH have also been investigated. A study done by Zetterling and colleagues looked at the level of consciousness of patients in relation to lactate and pyruvate levels in the brain. They showed that early (< 36 h of bleeding) interstitial pyruvate levels vary with the level of consciousness at admission in SAH patients, but measured no changes in lactate or the LPR. Moreover, conscious SAH patients had normal pyruvate levels (159–196 µM), but unconscious patients showed low or subnormal levels (102–131 µM) [54].

In patients with SAH, a change in blood viscosity over time due to hemoglobin degradation and its metabolites can obstruct the outflow of CSF and can cause hydrocephalus. Hydrocephalus is a significant medical complication and requires a shunt in 6–67% in SAH patients [63,64,65]. Wang and colleagues studied the biochemical differences between ventricular and intrathecal CSF and assessed the role of CSF lactate in shunt-dependent hydrocephalus after SAH [55]. They obtained intrathecal and intraventricular CSF from patients with a modified Fisher grade 3 and 4, on day 7 post SAH and found that only elevated intrathecal lactate (> 5.5 µmol/L) predicted the occurrence of hydrocephalus. After a multivariate analysis for clinical and CSF variables (total protein, ferritin, hemoglobin), they showed that elevated CSF lactate and intraventricular hemorrhage were independently associated with shunt-dependent hydrocephalus [55]. Another significant medical complication of SAH, pneumonia, has been also studied in association with lactate. A prospective study of 18 patients was conducted to observe the correlation between interstitial lactate and pneumonia after SAH. Pneumonia was defined as new infiltrate on a chest X-ray, with purulent sputum, and/or positive microbiological cultures. They found early (day 3) interstitial lactate levels were elevated in patients with bacterial pneumonia (median, 6.82 mmol/L) compared to those without pneumonia (median, 2.90 mmol/L) [36]. In SAH patients, neurogenic pulmonary edema (NPE) is a severe complication and contributes to unfavorable outcomes [66]. A retrospective study of 140 patients looked at the association between lactate and NPE defined as bilateral diffuse, alveolar infiltrates in chest radiographs on arrival in hospital. The study showed a correlation between elevated serum lactate levels (54 mg/dl versus 28 mg/dl) occurring within one hour of SAH and the early onset of NPE [56].

## 6. Conclusions

SAH causes devastating brain injury with broad repercussions for the patient and their family. A prognostic biomarker which is altered in the acute phase and can accurately predict secondary events and patient outcomes would be immeasurably beneficial, as clinical monitoring is of limited value in SAH patients who are often sedated or in a coma. Furthermore, the current severity grading scores are inadequate in the prediction of long-term outcomes and secondary events, and thus there is great need for a predictive biomarker. This review has focused on the cerebral metabolites lactate and pyruvate after SAH in an effort to summarize the current clinical research and evaluate the potential of these metabolites as an acute phase biomarker of prognosis after SAH. The fluctuations in the levels of lactate and pyruvate in cerebral fluids are reflective of changes in cellular energy metabolism and can be used to predict and measure metabolic crisis in patients who may not yet be symptomatic. Elevated levels of lactate and an increase in the LPR correlates with impending ischemia and neurological deficits, as well as poor patient outcomes and a high mortality rate. Similarly, they are associated with elevated ICP, hemorrhage extension, and secondary events such as hydrocephalus and pneumonia. Moreover, the changes in lactate and pyruvate levels can be measured from CSF and ISF, as well as more routinely accessible sources, such as blood.

Despite their potential as biomarkers for SAH outcomes, further studies are required to reach consensus about the clinical viability and accuracy of lactate and pyruvate as a predictive tool. Specifically, a threshold needs to be defined for what constitutes elevated levels after SAH and across different age groups. Moreover, the timing of measurement needs to be investigated to find an optimal predictive window of time, as well as determining which biofluid is the most clinically relevant, while still accurate and accessible. Ultimately, it may be that in the era of multi-omic’s approaches, lactate and pyruvate measurements will be integrated into a multimodal prognostic model to guide precision therapy for SAH and improve patient outcomes.

## Figures and Tables

**Table 1:** Clinical studies investigating cerebral lactate and pyruvate in subarachnoid hemorrhage.

Study Design	Sample Size	Age, y (±SD or Range) *	Sex	Sample (Method)	Relevant Results	Ref.
**R**	46	61.0 ± 10.7	18 M 28 F	CSF (LP and cisternal drain)	Elevated CSF pyruvate concentration is strongly associated with poor grade SAH (WFNS ≥ III).	[16]
**R**	55	55 ± 12	15 M 40 F	ISF (CMD)	Biochemical patterns of mitochondrial dysfunction (LPR > 30) in 29 patients, and cerebral ischemia (LPR > 30 and > 40) in 10 patients, including 6 patients who also demonstrated periods of mitochondrial dysfunction.	[29]
**R**	249	55 ± 11	102 M 47 F	Serum	Elevated admission serum lactate is predictive of mortality (3.5 ± 2.5 mmol/L vs. 2.2 ± 1.6 mmol/L).	[30]
**R**	105	59 ± 13	34 M 71 F	Serum	Early (24 h from admission), serum lactate elevation > 2.2 mmol/L (mean of 2.91 mmol/L) did not independently predict patient mortality and discharge (adjusted odds for Hunt and Hess scale, GCS, age and DCI).	[31]
**P**	20	60 (51–64)	2 M 18 F	ISF (CMD)	Elevated lactate levels and high LPR (51 ± 36) is correlated with delayed cerebral hypoperfusion (< 32.5 mL/100 g/min) in comatose patients with SAH.	[35]
**P**	18	52 ± 10.7	8 M 10 F	ISF (CMD)	Early (day 3) interstitial lactate levels are elevated in patients with bacterial pneumonia (median, 6.82 mmol/L) compared to those without pneumonia (median, 2.90 mmol/L).	[36]
**R**	285	55 (47–65)	96 M 189 F	Serum	Elevated serum lactate levels (≥ 2.1 mmol/L) in first 24 h after SAH are associated with increased risk of DCI and poor outcomes (mRS of 4–6 at 3 months).	[40]
**R**	145	62 ± 16.3	44 M 101 F	Serum	Serum lactate of > 1.1 mmol/L after 48 h of admission is the most accurate predictor of unfavorable neurological outcomes in terms of mRS at discharge.	[41]
**R**	33	61 (26–77)	11 M 22 F	CSF (EVD)	No association between elevated CSF lactate > 2.1 mmol/L (at 0–240 h post SAH measurement) and impaired circulation and clinical outcomes. However age (≥ 61 years) and coiling for treatment are significantly correlated with elevated lactate levels.	[42]
**P**	20	60 (32–83)	9 M 11 F	CSF (EVD)	CSF lactate in the first 12 days after SAH and an increased LPR on days 5–7 correlated with onset of cerebral vasospasm.	[44]
**R**	51	55 (44–64)	18 M 33F	CSF (EVD)	Elevated CSF lactate level (median, 3.2 mmol/L) within 10 days post-SAH correlates with intraventricular hemorrhage and unfavorable outcomes at discharge.	[45]
**P**	15	N/A	N/A	ISF (CMD)	Interstitial LPR > 30 is associated with decreased cerebral perfusion, but not with increased ICP of greater than 20 mmHg.	[50]
**R**	21	48 ± 15.9	14 M 7 F	ISF (CMD)	Elevated LPR (50.01 ± 24.79) correlates with increased mortality. Survivors had elevated lactate values (8.52 mmol/L vs. 5.89 mmol/L) compared to non survivors.	[51]
**R**	30	58.9 (28–84)	5 M 25 F	ISF (CMD)	Low CBF (< 28 mL/100 g/min), elevated ISF lactate (4.8 ± 2.2 mmol/L), and elevated LPR (32 ± 16) are early warning signs (day 0–3) of DCI before any clinical symptoms appear.	[52]
**R**	30	58.9 (28–84)	5 M 25 F	ISF (CMD)	Blood flow measurements and CMD sample monitoring on days 0–3 after onset of SAH showed elevated lactate levels 3.9 ± 2 mmol/L and low regional CBF in territory of the impending ischemia.	[53]
**P**	19	55 (46–73)	6 M 13 F	ISF (CMD)	Interstitial pyruvate levels vary with level of consciousness. Between 84 and 132 h after SAH, conscious SAH individuals had normal levels (159–196 µM) but in unconscious SAH patients, pyruvate levels remained low (102–131 µM).	[54]
**R**	28	55.4	13 M 15 F	CSF (intrathecal and intraventricular)	Patients with modified Fisher grades 3 and 4 have elevated intrathecal CSF lactate (> 5.5 mmol/L) on day 7 post SAH and is predictive of poor neurological outcomes and hydrocephalus requiring a shunt.	[55]
**R**	140	48 (47.3–65.5) (patients who developed NPE only)	48 M 92 F	Serum	Increase serum lactate levels (54.0 mg/dl), within one hour after SAH are associated with early onset of neurogenic pulmonary edema (NPE).	[56]
**P**	10	52.2 ± 5.0	7 M 3 W	ISF (CMD)	10 fold increase in lactate levels (baseline levels 100–200 µmol/L) along with increase in excitatory amino acids in SAH showed correlation with poor outcomes at the 3 months (GOS 1 to 3).	[33]
**P**	22	56 (47–68)	7 M 15 F	ISF (CMD)	High CMD tau protein was positively correlated with elevated levels of lactate (> 4 mmol/L) and also positively correlated with pyruvate, LPR, and poor functional outcome (mRS ≥ 4) 12 months after SAH after adjusting for disease severity and age.	[39]

* Age mentioned either in standard deviation (SD) or range. Abbreviations: R, retrospective; P, prospective; y, year; SD, standard deviation; M, male; F, female; SAH, subarachnoid hemorrhage; CMD, cerebral microdialysis; ISF, interstitial fluid; LP, lumbar puncture; GOS, Glasgow coma scale; LPR, lactate-pyruvate ratio; EVD, external ventricular drain; DCI, delayed cerebral ischemia; mRS, modified Rankin Score; CSF, cerebrospinal fluid; GCS, Glasgow coma scale; ICP, intracranial pressure; PCT, perfusion computed tomography; CBF, cerebral blood flow; NPE, neurogenic pulmonary edema; N/A, not available.
